# Psychometric properties of the Hungarian version of the eHealth Literacy Scale

**DOI:** 10.1007/s10198-019-01062-1

**Published:** 2019-05-16

**Authors:** Zsombor Zrubka, Ottó Hajdu, Fanni Rencz, Petra Baji, László Gulácsi, Márta Péntek

**Affiliations:** 1 Department of Health Economics, Corvinus University of Budapest, Fővám tér 8., 1093 Budapest, Hungary; 2 Doctoral School of Management, Corvinus University of Budapest, Fővám tér 8., 1093 Budapest, Hungary; 3 Department of Comparative Economics, Eötvös Loránd University, Egyetem tér 1-3, 1053 Budapest, Hungary; 4 Premium Postdoctoral Research Program, Hungarian Academy of Sciences, Nádor u. 7, 1051 Budapest, Hungary

**Keywords:** eHEALS, eHealth literacy, Item-response theory, Validation, Hungary, EQ-5D-5L, I10

## Abstract

**Background:**

We adapted the eHealth Literacy Scale (eHEALS) for Hungary and tested its psychometric properties on a large representative online sample of the general population.

**Methods:**

The Hungarian version of eHEALS was developed using forward–backward translation. For the valuation study, 1000 respondents were recruited in early 2019 from a large online panel by a survey company. We tested internal consistency, test–retest reliability and construct and criterion validity using classical test theory, as well as item characteristics using an item-response theory (IRT) graded response model (GRM).

**Results:**

55% of respondents were female, and 22.1% were ≥ 65 years old. Mean eHEALS score was 29.2 (SD: 5.18). Internal consistency was good (Cronbach’s *α* = 0.90), and test–retest reliability was moderate (intraclass correlation *r* = 0.64). We identified a single-factor structure by exploratory factor analysis, explaining 85% of test variance. Essential criteria for GRM analysis were met. Items 3 and 4 (search of health resources) were the least difficult, followed by items 5 and 8 (utilisation of health information), and then items 1 and 2 (awareness of health resources). Items 6 and 7 (appraisal of health resources) were most difficult. The measurement properties of eHEALS were not affected by gender, age, education or income levels. Female gender, older age, intensity of health information seeking, formal health education and visit at the electronic health-record website were associated with higher eHEALS scores, as well as best and worst self-perceived health states, BMI < 25 and participation at health screenings over the past year.

**Conclusions:**

The Hungarian eHEALS is a useful and valid tool for measuring subjective eHealth literacy.

**Electronic supplementary material:**

The online version of this article (10.1007/s10198-019-01062-1) contains supplementary material, which is available to authorized users.

## Introduction

Digital revolution is changing health care, both in technical and cultural terms. Health information is available without temporal and spatial constraints, and one-third of European citizens search the Internet for health information on a monthly basis [1]. In the digital era, we expect that being helped by technology, patients will eventually become their physicians’ partners in medical decision-making [2]. We also expect that being connected and informed, patients will be better empowered to take control of their health and demand better value [3], eventually leading to improved quality and reduced costs of health care, although some of these expectations have yet to be proven [4]. However, despite the promises of eHealth, some of the online information may be inaccurate or even harmful [5, 6]. With the digital transformation of patient education media, patients are becoming increasingly responsible for evaluating the accuracy and reliability of the information they find over the Internet, which assumes non-trivial skills: electronic health literacy (eHealth literacy).

Health literacy is defined as “the degree to which individuals have the capacity to obtain, process, and understand basic health information and services needed to make appropriate health decisions” [7], while according to the Lily model of Norman and Skinner [8], eHealth literacy covers a broader concept, encompassing traditional literacy (basic ability to read and comprehend written text), information literacy (the ability to find and use information), media literacy (the ability to think critically about media content and context), computer literacy (the ability to use computers for problem solving) and scientific literacy (understanding how knowledge is created with its aims, methods, limitations, and politics), in addition to traditional health literacy. eHealth literacy has been defined as “the ability to seek, find, understand, and appraise health information from electronic sources and apply the knowledge gained to addressing or solving a health problem” [8].

eHealth literacy can be viewed as a resource that enables the individual to achieve better subjective health status [9–11], lower risk of chronic disease [12], healthy lifestyle [13–16], or more health-conscious behaviour, such as regular participation at cancer screenings [17]. However, a widening digital divide has been demonstrated in the field of health care. Typically, patients, who consume most health-care resources, the elderly and the ones with low socioeconomic status or low education, lack the skills to utilise effectively electronic health information or services [12, 18, 19]. Electronic patient education media and services are frequently not tailored to the diverse needs of patients with varying levels of eHealth literacy [20].

To realise the societal and economic benefits of eHealth efficiently and fairly, we need to monitor and develop the level of eHealth literacy of people [21], design interventions appropriately, measure their impact, and take care that no one is left behind. eHealth literacy can be measured by objective, performance-based methods, which evaluate the results of tasks performed in simulated laboratory environments. These methods can be cumbersome and require considerable technical equipment; therefore, their main use is in exploratory research or the validation of self-rating instruments [22–26]. Self-rating instruments contain items about attitudes or behaviours related to the theoretical concepts of eHealth literacy and can be easily administered even in clinical environments. However, most self-rating instruments have shown limited correlation with objective measures; therefore at best, they can be viewed as the measures of subjective eHealth literacy [24, 27–30]. Indirect strategies aim to map the details of the frequency, sources, variability, etc. of online health information-seeking behaviour, to infer the level of eHealth literacy [1]. Mixed strategies combine standalone measures of the elements of eHealth literacy, for example measure separately traditional health literacy and computer literacy [31]. Among all measures, the eHealth Literacy Scale (eHEALS) [27] has been used most extensively. Rooted in Albert Bandura’s social cognitive theory and self-efficacy theory [32], eHEALS is a subjective measure of eHealth literacy, showing little correlation with traditional health literacy [25, 33, 34] or the objective high-level skills of searching and critically evaluating health-related online information. Rather, eHEALS can be viewed as a self-efficacy-related measure of eHealth literacy. eHEALS was developed and validated in 2006 on Canadian adolescents during an RCT measuring the efficacy of an online anti-smoking intervention [27]. Subsequently, the reliability and validity of eHEALS has been shown in a representative sample from the general population [12], in chronic patients [35, 36], people with low socioeconomic status [37], the elderly [38] as well as interculturally [39], in several languages [12, 23, 40–45] and in phone-based surveys [12, 46]. In addition to classical test theory methods, the psychometric properties of eHEALS have been tested using the methods of item-response theory (IRT) [35, 36, 41]. However, to our knowledge, eHEALS has not been adapted in countries of the Central and Eastern European region. Therefore, our aim was to measure the eHealth literacy and test the psychometric properties of eHEALS in a large online representative sample of the Hungarian population.

## Methods

### Sample

A representative, cross-sectional, Internet-based survey was carried out in early 2019. Ethical approval was obtained by the Hungarian Medical Research Council (ID: 47654-2/2018/EKU). Recruitment and data collection were carried out from a large online panel by a survey company, Big Data Scientist Kft. The target sample size was 1000, using quotas to ensure the representativeness of the sample by gender, age, educational level, and type and region of residence between the age of 18 and 65 years. We aimed to obtain a reasonable sample over the age 65 years.

Participation in the survey was completely voluntary and anonymous. Respondents were informed about keeping the privacy of their personal information and that results would be used solely for scientific purposes. Respondents needed to provide their informed consent at the start of the survey and further confirm their consent to participate at the end of the survey. From completers of the survey, a random sample of 50 was selected for repeated administration of selected questions 2 weeks after the initial participation.

### Questionnaire

We measured eHealth literacy as part of a larger survey titled “Patient Experiences in Healthcare” also exploring shared decision-making (SDM) and patient-reported experience measures (PREMS) of outpatient care in Hungary.

Data were collected on the social-demographic characteristics of all respondents (such as age, gender, highest level of completed education, marital status, and current employment status), the household of the respondent (size, monthly net income), and the place of residence (type of residence, geographical region). We established the following risk groups based on socio-demographic status: age ≥ 65 years, education ≤ 8 years, household income per capita in first quintile of the sample [47].

The health status of respondents was measured by the three questions of the Minimum European Health Module (self-perceived health, chronic morbidity, and the Global Activity Limitation Indicator) [48] and the Hungarian version of the EQ-5D-5L questionnaire [49]. We also asked whether respondents considered their lifestyle healthier or less healthy than others, and whether they were informal caregivers. We queried about weight and height, smoking status, frequency of alcohol consumption, and participation in any health screening test over the past 12 months. We considered the following lifestyle parameters as having a health-related risk factor: body mass index (BMI) ≥ 25 [50], current smoker status [51], alcohol consumption ≥ 3 times a week [52], no exercise at all [53], and no participation at any health screening over the past 12 months [54]. Also, we recorded whether respondents had any formal health education. Regarding the intensity of health information seeking, we asked about the frequency (at least weekly, several times a month, monthly, bimonthly, several times a year, none), goals (solve health problem without involvement of health professional, decide if consultation is needed with health professional, prepare for visit with health professional, check information after consultation with health professional, other) and primary sources used (health professional, laypeople, Internet informational sites, Internet forums or social media, printed materials, television/radio programmes or advertisements in any media). We also asked, for whom the respondent was looking for health information. As a proxy for self-reported general health literacy, we asked at what level respondents understand health-related information (difficulties despite assistance, need assistance to understand, understand without assistance, understand so well that others seek help from the respondent). Electronic health records (EHR) were introduced universally in Hungary from late 2017, so we asked whether respondents were aware of or visited the EHR website as a proxy for eHealth awareness.

### eHEALS

eHEALS consists of eight items, each scored on a five-point Likert-scale with options ranging from “strongly disagree” to “strongly agree” [27] Levels of the items are added for a total score ranging between 8 and 40, with higher scores indicating greater skill. Items 1 and 2 are related to awareness, items 3 and 4 to searching and items 6 and 7 to appraisal of health resources, and items 5 and 8 are related to utilisation of health information. The original English and adapted Hungarian versions are included in the Supplementary Material S1. The original questionnaire has a single-factor structure, good internal consistency, and modest test–retest reliability [27]. The questionnaire contains two supplementary items to assess the general interest of the respondent in using Internet for health information: usefulness and importance, which are not calculated in the total score.

Several Hungarian translations of eHEALS were made independently and discussed by native Hungarian researchers involved in the study. The consolidated version was backward translated by an independent professional agency and evaluated by members of the research team. In five items of eHEALS, the questions referred to *“*health resources”, which could be translated as “piece of health information” or “source of health information”. We contacted the authors of eHEALS to clarify the original intent and used the “source of health information” meaning in the final version. We piloted the survey questionnaire involving five respondents, and based on the feedback from cognitive de-briefing, the wording of item 6 was refined. In this item, Hungarian words for “skills” could refer to professional skills, practical skills, or knowledge. In the final version, we chose the word with the closest meaning to “knowledge”.

### Statistical analysis

We used descriptive statistics to characterise the sample and explore eHEALS distribution, as well as eHEALS mean scores and their dispersion by sample subgroups. Mean scores by subgroup were compared by *t* test for binary groups and ANOVA for multiple categories, and standard deviations were compared by Levene’s robust test [55]. We tested internal consistency by calculating Cronbach’s *α* for the whole test and compared *α* statistics for key socio-demographic risk groups using the Feldt test [56] to analyse measurement bias. We provided item-test correlation values for each item. Test–retest reliability was calculated by the intraclass correlation coefficient.

We checked construct validity via exploratory factor analysis (EFA), by performing principal factor analysis of the polychoric correlation matrix of the ordinal test items [57]. We considered factors with eigenvalues ≥ 1 relevant for the test structure. We tested adequacy of our sample for factor analysis by the Bartlett test for sphericity and the Kaiser–Meyer–Olkin statistic. Although we did not apply other validated tests of eHealth literacy or its related constructs, we tested convergent validity by measuring the level of association between eHEALS scores and health information-seeking behaviours. We expected significant positive association with the frequency of health information-seeking behaviour, the preference for, the importance and usefulness of using the Internet for health information, the level of health-related knowledge of respondents (formal health education and self-reported level of understanding) as well as checking the EHR website.

We tested concurrent validity by examining the association of eHEALS with health status and health-related lifestyle variables. We expected positive association with health status and negative association with health-related risk factors. Discriminant validity was examined by testing the association of eHEALS with socio-demographic variables. We expected that eHealth literacy is standalone construct and shows only moderate association with age, education, or income level. For measuring associations with eHEALS, we calculated point-biserial correlation for dichotomous items (coefficient: *r*_bs_), Pearson correlation for continuous variables (coefficient: *r*), and polyserial correlation for polytomous ordinal items measuring an underlying construct (coefficient: *r*_ps_).

We also applied item-response theory (IRT) models to characterise the test items. IRT assumes that test results depend on the interaction of the measured latent trait of respondents as well as the characteristics of the test items. Both dichotomous or polytomous test items can be described by their level of difficulty (the latent trait level where the probability of scoring an item is 0.5) and discrimination power (differences in the latent trait levels between individuals with different item scores), as well as easiness of guessing [57].

For polytomous test items, parametric IRT models can estimate a difficulty and a discrimination parameter. The rating-scale model (RSM) assumes equal difficulty levels between items, and the same discrimination parameter for all items. The partial-credit model (PCM) assumes varying difficulty levels, but the same discrimination parameter of items. The general partial-credit model (GPCM) and graded response model (GRM) allow different difficulty levels as well as discrimination properties per item, but use different logit function models for the estimation. These models make no assumptions about guessing. The latent trait (eHealth literacy, denoted with “theta”) is assumed to follow a standard normal distribution with a mean value of 0 and standard deviation of 1. We compared four polytomous IRT models and selected the optimal one based on the lowest Akaike’s information criterion (AIC) values. We computed difficulty and discrimination parameters for each item and plotted the test characteristic curve (TCC) and the item-information functions (IIF). The TTC plots total test scores, while the IIF plots the information function of each item against the latent trait levels. The TCC shows how the test scores and the measured latent trait relate to each other and what is the measurement range of the instrument. The information function is inversely related to the variance of latent trait estimates. At a given latent trait level, greater information function values indicate greater precision of the estimate [57]. Item-information functions indicate at what latent trait levels each item contributes to the overall information provided by the test. IRT analyses were performed with the Stata 14.2 statistical software package and the ltm package of R using identical Gauss–Hermite quadrature integration method for log likelihood with 21 integration points [58, 59].

## Results

### General properties

**Fig. 1:**
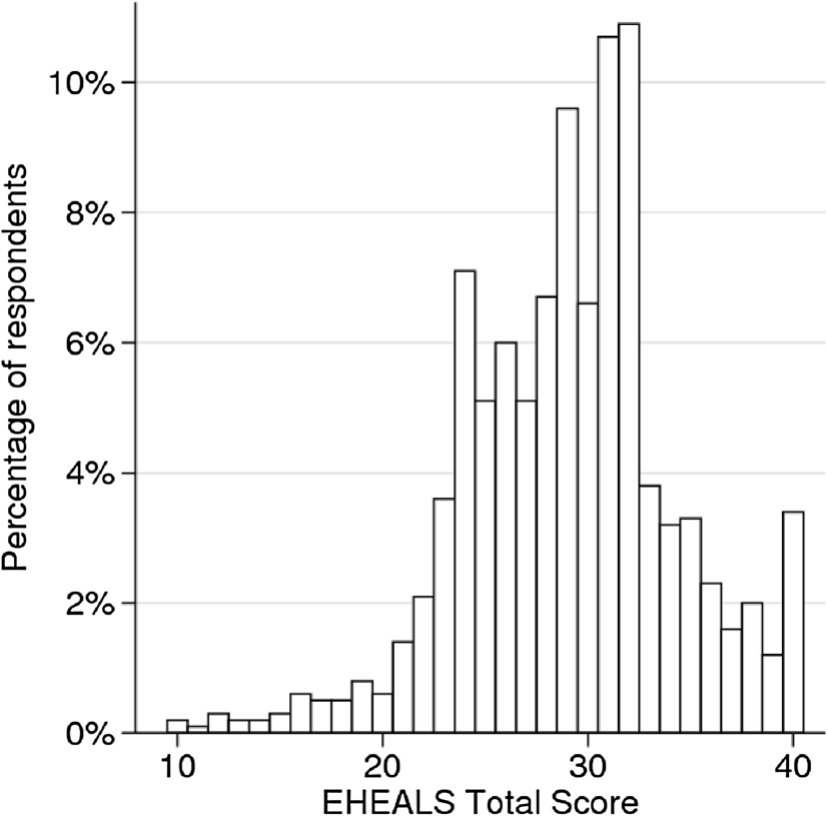
Distribution of eHEALS scores

The survey was completed by 1000 respondents. The sample was similar to the general population in most characteristics, with slight shift towards individuals with higher education levels (Table 1). The mean age was 46.3 years (SD: 17.7 years, range: 18–90 years), and 55% of respondents were female. Median time to complete eHEALS was 66 s. Completion time was between 31 and 190.5 s for 90% of respondents. Response time did not influence the eHEALS scores. The distribution of eHEALS was left skewed with reasonably good fit on the normal curve for scores between 20 and 39, and a heavy left tail at scores below 20 and a peak at score 40 (Fig. 1). The mean eHEALS score was 29.16 (SD: 5.18). The Bartlett test for sphericity (*p* < 0.001) and Kaiser–Meyer–Olkin statistic of 0.89 suggested that the data were suitable for factor analysis. EFA based on the polychoric correlation matrix provided eigenvalues of 5.18, 0.61 and 0.28 for factors 1, 2 and 3, respectively, which indicated a single-factor structure. The inter-item polychoric correlation coefficients ranged between 0.41 and 0.93, and factor loadings ranged between 0.67 and 0.92. The single factor explained 85% of the variance. Cronbach’s *α* was 0.90, suggesting good internal consistency. Item-test correlation coefficients ranged between 0.71 and 0.82 (Table 2). The intraclass correlation between first and second administration was 0.64, indicating moderate stability over time.

**Table 1:** Sample characteristics

	First administration		Second administration		General population
	(*N*)	(%)	(*N*)	(%)	(%)
Total	1000	100.0	50	100.0	100.0
Gender					
Female	550	55.0	22	44.0	53.4
Male	450	45.0	28	56.0	46.6
Age (years)					
18–24	118	11.8	4	8.0	10.6
25–34	198	19.8	8	16.0	16.9
35–44	191	19.1	12	24.0	18.8
45–54	125	12.5	9	18.0	15.5
55–64	147	14.7	7	14.0	17.6
65 +	221	22.1	10	20.0	20.6
Education					
Primary	341	34.1	23	46.0	51.0
Secondary	363	36.3	11	22.0	31.3
Tertiary	296	29.6	16	32.0	17.7
Type of residence					
Capital	213	21.3	13	26.0	18.1
Urban	557	55.7	21	42.0	51.9
Rural	230	23.0	16	32.0	30.0
Geographical region					
Middle	348	34.8	18	36.0	30.0
East	353	35.3	21	42.0	39.6
West	299	29.9	11	22.0	30.4

^a^General population over 18 years of age, 2011 European Census Data [72]

**Table 2:** Psychometric characteristics of eHEALS by item

Item	Wording	Item-test corr.^a^	Factor loading	Level difficulty^b^				Item discr.^c^
				Disagree	Undecided	Agree	Strongly agree	
1	I know what health resources are available on the Internet	0.71	0.73	– 3.04	– 2.28	– 0.25	1.54	2.05
2	I know where to find helpful health resources on the Internet	0.79	0.82	– 2.53	– 1.69	– 0.29	1.42	2.63
3	I know how to find helpful health resources on the Internet	0.82	0.88	– 2.58	– 1.77	– 0.59	1.03	3.67
4	I know how to use the Internet to answer my questions about health	0.80	0.85	– 2.83	– 1.96	– 0.75	0.99	3.33
5	I know how to use the health information I find on the Internet to help me	0.81	0.84	– 2.74	– 1.91	– 0.53	1.21	2.97
6	I have the skills I need to evaluate the health resources I find on the Internet	0.77	0.75	– 2.46	– 1.18	0.32	1.74	2.01
7	I can tell high quality health resources from low-quality health resources on the Internet	0.71	0.67	– 2.94	– 1.70	– 0.12	1.68	1.65
8	I feel confident in using information from the Internet to make health decisions	0.79	0.78	– 2.79	– 1.61	– 0.22	1.37	2.20

^a^Item-test correlation

^b^Level difficulty: the latent trait level (distance from mean in standard deviations), where the probability of scoring an item is ≥ 0.5

^c^Item discrimination

### Association with socio-demographic variables

Female respondents had on average 0.7 points higher eHEALS scores than males (*p* = 0.040) and had significantly smaller SD values (*p* = 0.0037). Mean and SD values of the socio-demographic risk groups (age over 65 years, low education and low income) did not differ from the rest of the sample, with the exception of 0.98 lower mean score for ≥ 65-year-old respondents compared to younger ones (*p* = 0.013) (Table 3). EFA indicated single-factor structure within all socio-demographic risk groups. Cronbach’s *α* values ranged between 0.868 and 0.917, with significant differences only between males and females (*p* = 0.0002). The correlation of eHEALS scores with gender (*r*_pb_ = − 0.065, *p* = 0.040) and age were statistically significant, but weak.(*r* = − 0.113, *p* = 0.0004). The association of eHEALS scores with low education and low-income status was not significant. Furthermore, we found no difference between the urban and rural populations.

**Table 3:** Mean eHEALS scores and standard deviation by key sample subgroups

Domain	Variable	Level	*N*	eHEALS	
				(Mean)	(SD)
Socio-demographic characteristics	Gender***	Female	550	29.5	4.76
		Male	450	28.8	5.64
	Age*	< 65	779	29.4	5.13
		≥ 65	221	28.4	5.29
	Household income per capita	1st quintile within sample	167	29.8	5.29
		2–5th quintile within sample	655	29.1	4.71
	Education	≤ 8 years	86	28.5	5.06
		> 8 years	914	29.2	5.19
Health status and health-related lifestyle	BMI*	BMI < 25	386	29.8	4.75
		BMI ≥ 25	614	28.7	5.39
	Current smoker	No	706	29.1	5.20
		Yes	294	29.4	5.15
	Alcohol intake	≥ 3 times/week	857	29.2	5.11
		< 3 times/week	143	28.9	5.60
	Exercise**	None	541	29.4	4.78
		Some	459	28.9	5.61
	Health screening in past 12 months*	Yes	463	29.8	4.92
		No	537	28.6	5.35
	Self-rated lifestyle*	Healthier than others	221	30.2	4.99
		As healthy as others	600	28.9	5.08
		Less healthy than others	179	28.6	5.56
	Self-perceived health***	Very good	124	30.7	5.69
		Good	471	29.0	5.04
		Fair	323	28.9	4.83
		Bad	77	28.4	6.02
		Very Bad	5	34.2	5.67
	Informal caregiver status	Yes	127	29.5	4.76
		No	873	29.1	5.24
Health information seeking	Health information seeking over the past 12 months***	Weekly	152	31.5	4.49
		Several times/month	263	29.7	4.86
		Once in a month	166	29.0	4.45
		Bimonthly	94	28.9	4.53
		Several- times/year	270	28.6	5.06
		None	55	23.7	7.31
	Primary source of health information*	Physician/health professional	417	29.3	4.83
		Layperson (friends, relatives)	38	27.3	6.31
		Internet informational sites	317	29.8	4.51
		Internet (social media, forums)	89	29.1	4.67
		Printed materials	27	30.8	5.51
		Television/radio programmes	27	31.9	5.13
		Advertisements (any media)	26	29.5	5.19
	Have visited the EHR website? ***	Not aware of the site	761	28.9	5.36
		Aware, but haven’t visited	192	29.7	4.43
		Visited	47	30.5	4.81
	Formal health education*	Yes	74	30.6	5.74
		No	926	29.0	5.12
	Level of understanding of health information***	Difficulties despite assistance	6	21.7	8.78
		Needs assistance	174	26.3	5.65
		Understands well	704	29.3	4.64
		Others seek help from him/her	116	33.0	5.53
Total sample			1000	29.2	5.18

*BMI* Body Mass Index, *EHR* electronic health-record

*Significant difference of means (ANOVA, *p* < 0.05)

**Significant difference of standard deviations (Levene’s robust variance test, *p* < 0.05)

***Significant difference of means and standard deviations

### Association with health information seeking

We found significant relationship between the frequency of health information seeking and eHEALS scores. Those who reported seeking health information on a weekly basis had on average 1.9–2.9 higher scores compared to the ones who sought less frequently (*p* < 0.001), and 7.8 higher compared to ones who had not searched for health information over the past 12 months (*p* < 0.001). The primary source of health information also influenced eHEALS scores. Compared to respondents whose primary source of health information was a health-care professional, ones who sought information primarily from laypeople (friends, relatives with no health education) had on average 2 points lower eHEALS scores (*p* = 0.015), while ones who sought information primarily from television or radio programmes had 2.6 points higher eHEALS scores (*p* = 0.006). Interestingly, not those respondents who indicated informational websites as their primary source of health information had the highest eHEALS scores. Those who had already visited the EHR website had 1.6 points higher eHEALS scores compared to ones who had not even heard about it (*p* = 0.041). Respondents with formal health education also had 1.6 points higher eHEALS scores compared to the rest of the sample (*p* = 0.011). There were 22 respondents in the sample with tertiary education and formal health education. Their mean eHEALS scores were 3.8 higher compared to the rest of the sample (*p* = 0.001). Among these respondents, the ones who reported seeking information on a weekly basis (*n* = 6), had 7 points higher mean eHEALS scores than the rest of the sample (*p* < 0.001). Compared to respondents who reported good understanding of health information, those who had difficulties despite help (*n* = 6) reported 7.6 points lower (*p* < 0.001), while those who needed help 3 points lower (*p* < 0.000), and those whose assistance was sought by others 3.7 points higher (*p* < 0.001) eHEALS scores (Table 3). The types of goals of seeking health information did not influence the eHEALS scores. However, a greater intensity of health information seeking was associated with higher eHealth literacy: eHEALS scores positively correlated with the number of goals reported by respondents (*r* = 0.18, *p* < 0.001), with the frequency of seeking health information (*r*_ps_ = 0.29, *p* < 0.001), and number of health information sources used (*r* = 0.14, *p* < 0.001). Furthermore, visiting the EHR website (*r*_ps_ = 0.11, *p* = 0.009), having formal health education (*r*_bs_ = 0.08, *p* = 0.011) as well as the subjective level of understanding health information (*r*_ps_ = 0.41, p < 0.001), the perceived usefulness of the Internet for health information (*r*_ps_ = 0.49, *p* < 0.001), and perceived importance of the Internet for health information (*r*_ps_ = 0.48, *p* < 0.001) were also positively associated with eHEALS scores.

### Association with health status and health-related lifestyle risks

Mean eHEALS scores were 1.19 lower for respondents with BMI≥25 compared ones with BMI < 25 (*p* < 0.001), and 1.12 lower for those who did not participate in any health screening during the past 12 months compared to the ones who did (*p* = 0.001). Other health-related lifestyle factors did not influence the eHEALS scores. The respondents who reported a healthier lifestyle compared to others had 1.4 points higher mean eHEALS scores compared to the ones who reported to living as healthily as others. The relationship with self-perceived health had a U-shape. Respondents reporting very good or very bad health had 1.8 (*p* = 0.001) and 5.3 (*p* = 0.025) higher mean eHEALS scores compared to the ones reporting fair health. Informal caregiver status did not influence eHEALS scores, nor having a chronic disease or the level of disease burden over the past 6 months. Respondents reporting any current problems with mobility, self-care, or usual activities on the EQ-5D-5L had, respectively, 1.3 (*p* < 0.001), 1.4 (*p* = 0.017), and 1.0 (*p* = 0.007) points lower eHEALS scores compared to the ones without problems. Although having any pain/discomfort or anxiety/depression problems (EQ-5D-5L) was not associated with eHEALS scores, the six patients reporting extreme levels of pain/discomfort and the five patients who reported extreme problems of anxiety/depression had 3.8 (*p* = 0.076) and 5.1 (*p* = 0.028) higher eHEALS scores compared to those without problems, respectively. The point-biserial correlation of eHEALS with health-related lifestyle risk parameters of high BMI (*r*_pb_ = − 0.11, *p* < 0.001) and non-participation at screenings (*r*_pb_ = − 0.11, *p* < 0.001) was weak. After artificially dichotomising self-reported health status to very good (n = 124) vs the rest of the sample or very bad (*n* = 5) vs the rest of the sample, point-biserial correlations [*r*_pb_ = 0.07 (*p* = 0.029) and *r*_pb_ = 0.11 (*p* < 0.001), respectively] indicated a weak relationship with eHEALS.

### Item-response theory analysis

From the estimated RSM (AIC:15645), the PCM (AIC:15556), the GPCM (AIC: 15349), and the GRM (AIC: 15146) models, we selected the GRM model based on the lowest AIC value. The essential criteria set by Linacre [60] for Rasch models were well met in terms of sample size, item category distribution, and monotonous advancement of measurements between each category. With the exception of one response category (“strongly disagree” in item 4), there were over ten respondents in each response category, and categories were monotonously ordered by theta levels. However, as outfit statistics are not applicable for the GRM [61], we tested model fit using the global likelihood ratio method. Initially, we fit a restricted model with a common discrimination parameter of 2.425 for all items (log-likelihood = − 7607.8, AIC: 15281) and then a flexible model allowing discrimination parameters to differ by item (log likelihood = − 7533.1, AIC: 15146). The likelihood ratio (LR) test suggested that the unrestricted model with item-specific discrimination parameters fit better our data (*χ*_( *df* = 7)_^2^ = 149.3, *p* < 0.001).

**Fig. 2:**
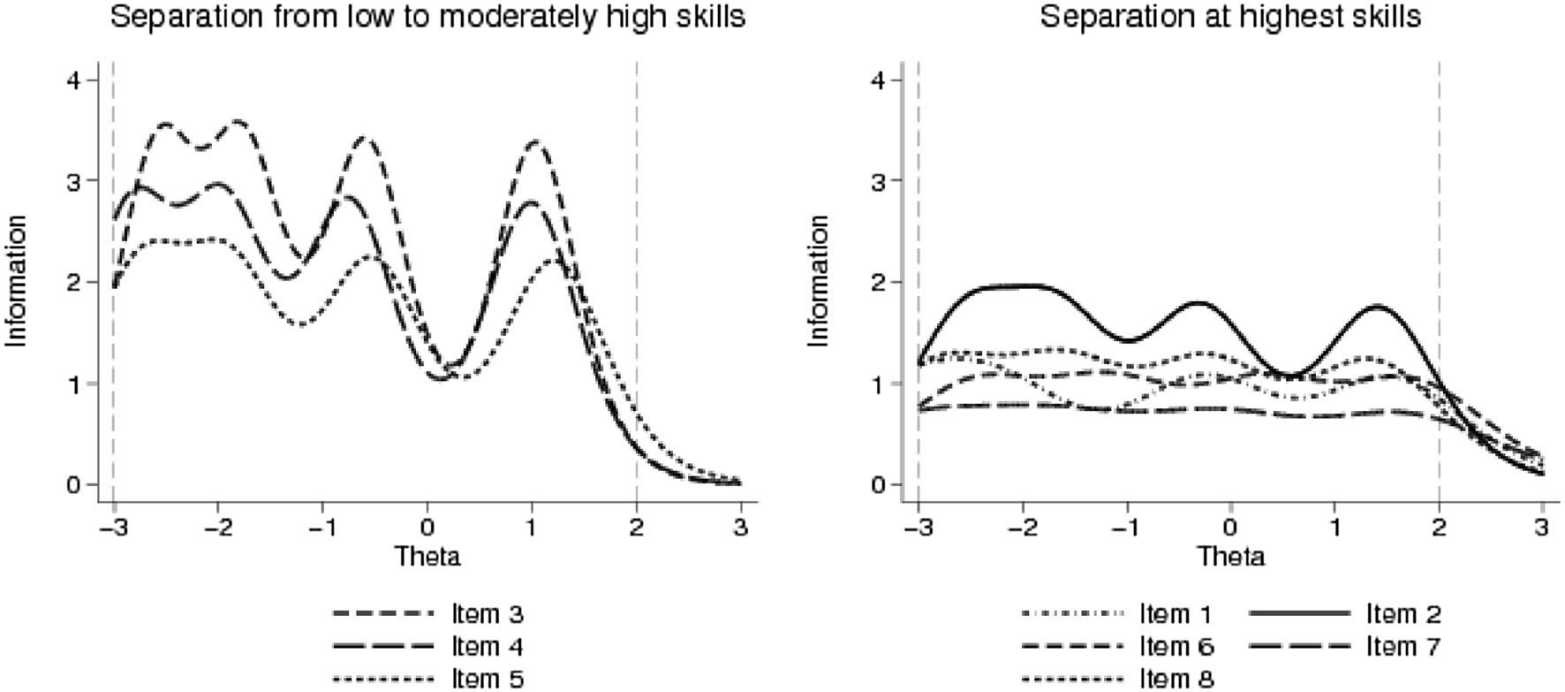
Item-information functions. Theta denotes the latent trait (standardized eHealth literacy). The information function is inversely related to the variance of the latent trait estimates

**Fig. 3:**
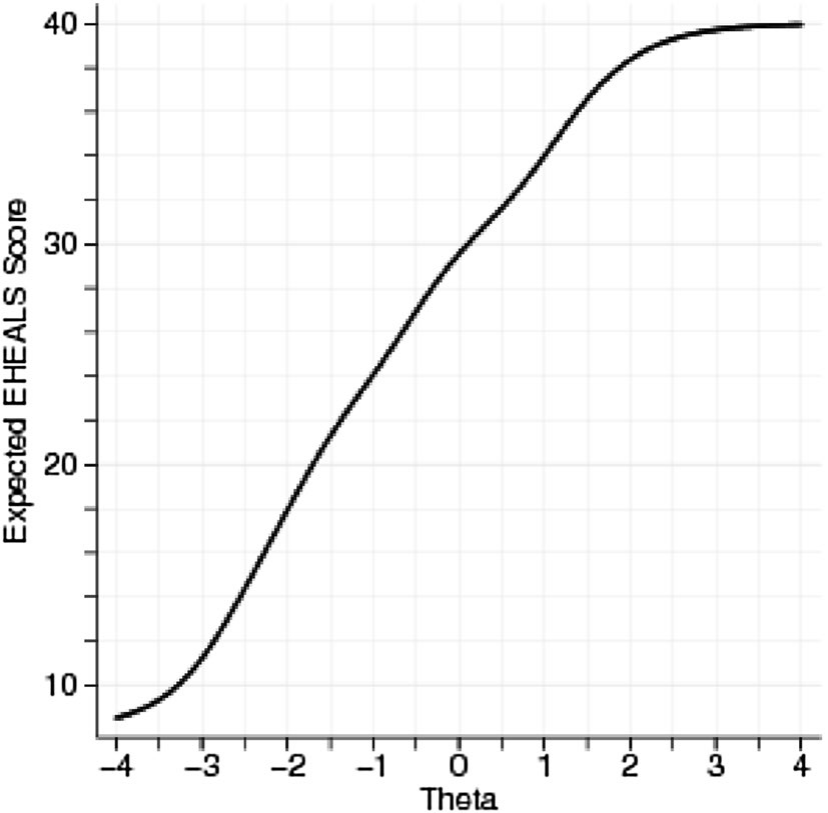
Test characteristic curve for the total eHEALS score. Theta denotes the latent trait (standardized eHealth literacy)

The difficulty and discrimination levels of each item are displayed in Table 2. Difficulty levels are expressed in standard deviations of *“theta”.* (Example for interpretation: individuals with eHealth literacy levels 1.48 SD above the mean are most likely to score “Strongly agree” on item 1). Items 6 and 7 (appraisal of health resources) had the greatest difficulty levels, followed by items 1 and 2 (awareness of health resources), and then by items 8 and 5 (utilisation of health information). The difficulty level was lowest for items 3 and 4 (searching of health resources). The discrimination power was greatest for the least difficult items 3 and 4, while items 6 and 7 had the weakest discrimination power. The item-information functions (Fig. 2) showed that items 3, 4, and 5 provide most information about respondents from the lowest levels up to moderately high levels of eHealth literacy, while items 1,2, 6, 7, and 8 provide most information about respondents with the highest skill levels. The test characteristic curve (Fig. 3) showed that eHEALS scores provide a near-linear measure of the latent trait of subjective eHealth literacy between − 3 (means eHEALS score = 11) and + 1.8 (mean eHEALS score = 38) standard deviations, providing more information about respondents with lower skill levels. The eHEALS score of 30 (29.7) corresponds best with the average subjective eHealth literacy level (*θ* = 0). On average, eHEALS scores 6 points above the mean indicate 1 SD lower than average, while 6 points above the mean indicate ~ 1.3 SD higher than average subjective eHealth literacy.

## Discussion

According to our knowledge, our study is the first to measure subjective eHealth literacy on a large representative online sample of the general population in Central–Eastern Europe. We tested the psychometric properties of the Hungarian version of eHEALS using methods of classical and modern test theory. A key strength of our study is that our sample containing 1000 respondents was similar to the general population in terms of age, gender, type, and geographical region of residence, involving 22.1% respondents above 65 years of age.

In our sample mean eHEALS score of 29.2 (SD: 5.18) was in the higher range among studies conducted in adult online populations. Mean scores were 23.4 in Japan [15], 26.7 in a Swiss population [41], 28.7, 29.2, and 30.5 among older online adults in New Zealand, UK, and the USA, respectively [39], and 30.3 among US chronic patients [35].

While other studies generally demonstrated lower eHealth literacy levels among older individuals and ones with low education or low income [12, 18, 37, 62], we found small, but statistically significant differences of eHEALS scores between males and females, as well as older (≥ 65) and younger adults, but no differences between individuals with low education or low income and the rest of the sample. The internal consistency was high in all socio-demographic risk groups. These results indicate good discriminant validity of eHEALS measuring a distinct characteristic only minimally explained by socio-demographic variables. However, our participants were regular Internet user members of an online panel, which may be more homogeneous compared to a true random sample from the general population, which is a limitation of our study.

The Hungarian version of eHEALS had good internal consistency (*α* = 0.90), similar to that of the original scale (*α* = 0.88) [27], as well as results of the Dutch (*α* = 0.93) [23], the Italian (*α* = 0.89) [41], and the Japanese (*α* = 0.93) [43] adaptation studies involving adult populations.

As in other studies, test–retest reliability of eHEALS was moderate (intraclass correlation *r* = 0.64) in our sample. From immediate to 6-month readministration sessions, the intraclass correlation coefficients of eHEALS ranged between 0.68 and 0.49 at its conception [27]. The test–retest reliability of the Japanese eHEALS scale was *r* = 0.63, [43], and after 30 days readministration, it was higher (*r* = 0.78) in the Spanish validation study [44].

We identified a similar single-factor structure as the developers of the original scale [27] and several validation studies [23, 35, 42, 44]. However, other studies identified a two-factor structure involving items 6 and 7 (appraisal of health resources) [41, 63] or items 5–8 (appraisal of health resources and utilisation of information) in a second factor [45, 64]. Studies in older adults also proposed a three-factor structure separating dimensions of awareness, skills, and evaluation [39, 46].

IRT analysis of eHEALS suggested that differences in the difficulty level of items might mimic a two-factorial structure of the otherwise unidimensional construct [41]. Our IRT analysis using a GRM model [65] also supports this view. The threshold values of “agree” or “strongly agree” responses indicated that items 6 and 7 (appraisal of health resources) had greatest difficulty levels, followed by items 1 and 2 (awareness of health resources), and then by items 5 and 8 (utilisation of health information). Items 3 and 4 (search of health resources) had the lowest difficulty level. These findings are in-line with recent studies observing real health information-searching behaviours: individuals use heuristics to select health information from search engine hits and the thorough checking of the credibility and quality of information sources is often skipped even by individuals with high reported levels of eHealth literacy [25, 66]. Other studies using IRT models identified different item orderings based on their difficulty levels. These studies involved smaller samples using RSM models [41, 67] or PCM models [39, 46]. We selected the GRM model based on lower AIC values than those of the RSM, PCM, or GPCM models. Also, our flexible model demonstrated better fit than a model assuming common item difficulty. Among all studies applying IRT analyses of eHEALS, to our knowledge our study had the greatest sample size (*n* = 1000), which is adequate for accurate parameter estimates in most GRM applications [68]. Furthermore, our analysis met essential criteria set by Linacre in terms of sample size requirements by item category, as well as category distributional properties and monotonicity of measurements, thereby supporting the reliability of our parameter estimates.

The associations of eHEALS with variables related to health information seeking were in line with our expectations and suggested a weak convergent validity of eHEALS with quasi-objective measures of health literacy (formal health education), eHealth awareness (visiting the EHR website), and the intensity (frequency and number of sources) of information seeking. Other studies demonstrated the association of eHealth literacy with the frequency of Internet use [12, 18, 23] and the number of health information sources [43] as well as the willingness to adopt EHRs [69], but the correlation was low with objective performance in eHealth literacy tests [23, 25, 66]. Overestimation of skills is a general feature of subjective computer literacy measures [70], while individuals with high eHealth literacy in real-life settings may have low motivation to excel in objective performance tests [25, 66]. These factors both weaken the association between objective and subjective health literacy measures.

The association of eHEALS scores with the subjective level of understanding health information as well as perceived usefulness and perceived importance of the Internet indicated a moderate convergent validity with subjective constructs related to eHealth literacy. However, preference of the Internet as a source of health information was not associated with higher eHealth literacy scores. Although informational websites were mentioned most frequently after health-care professionals as the primary source of health information, eHealth literacy levels were highest among the ones who prefer printed materials and tv/radio programmes. Interestingly, the five individuals who indicated Internet forums, laypeople, or advertisements among their top three sources of health information had higher mean eHEALS scores (32.6) compared to the sample average.

Probably, the most important question from public health or health economic perspective is whether subjective eHealth literacy is associated with better health outcomes, healthier lifestyle, better satisfaction with care, or more adequate and efficient utilisation of resources. Some studies demonstrated the association of higher eHealth literacy levels with better subjective health in chronic patient populations [9, 10], healthier lifestyle in terms of sleep, exercise and nutrition among college students [16], and with exercise and balanced diet [15], as well as participation in colorectal cancer screening [17] among Japanese Internet users. Similar to these findings, we found positive, albeit weak association of eHEALS scores with BMI < 25 and participation at health screenings. In addition to the weak association of eHEALS scores with the best ratings of self-perceived health, individuals with worst self-perceived health states also had higher than average eHEALS scores. Probably, in addition to the most health-conscious individuals, the most desperate ones also develop their eHealth literacy skills in search of relief for their symptoms. Altogether, in line with our prior expectations, eHEALS scores were positively associated with better subjective health or healthier lifestyle. The significant, albeit weak correlation suggested modest concurrent validity of eHEALS in terms of its association with the health status of the general population.

The concept of eHealth literacy is connected with self-efficacy [27, 32], a predictor of change in numerous health behaviours [71]. The convergent validity of eHEALS was not tested in relation to health-related self-efficacy measures which is a limitation of our study and an area of future research. A further area of research is whether subjective eHealth literacy in combination with measures of objective health literacy and other health psychology constructs may be useful in identifying segments of the population who are particularly susceptible for the benefits or potential risks of digital health.

## Conclusion

eHEALS showed favourable psychometric properties on a large, representative online sample of the Hungarian population. The internal consistency of the scale was good, while the test–retest reliability was moderate. We identified a single-factor structure with different item difficulty levels. According to IRT analysis using a graded response model, items related to search of health resources were the least difficult, followed by the ones related to the utilisation of health information and then the awareness of health resources. Items related to the appraisal of health resources were most difficult. The measurement properties of eHEALS were not affected by gender, age, education, or income levels. Content validity was similar to previous studies: eHEALS scores showed moderate correlation with subjective factors, and significant, but weak correlation with quasi-objective factors related to eHealth literacy. Also, eHealth literacy showed weak positive association with BMI < 25 and participation at screenings, as well as with the best and worst subjective health states. Altogether, eHEALS is an easy-to-administer and valid tool for measuring subjective eHealth literacy, while its properties predicting better health outcomes or more efficient use of health-care resources have yet to be determined in future studies.

## Electronic supplementary material

Below is the link to the electronic supplementary material.

Supplementary material 1 (PDF 143 kb)
